# Synthesis of 2- and 7- Substituted C_19_ Steroids Having a 1,4,6-Triene or 1,4-Diene Structure and Their Cytotoxic Effects on T47D and MDA-MB231 Breast Cancer Cells

**DOI:** 10.3390/molecules15064408

**Published:** 2010-06-21

**Authors:** Minwoo Kim, Eunsook Ma

**Affiliations:** College of Pharmacy, Catholic University of Daegu, Hayang, 712-702, Korea

**Keywords:** breast cancer, T47D cell, MDA-MB231 cell, 2-substituted 1,4,6-androstatriene-3,17-dione, 6α-hydroxy-7β-substituted-1,4-androstadiene-3,17-dione

## Abstract

2-Chloro-, 2-bromo- and 2-azido-1,4,6-androstatriene-3,17-diones were synthesized from 1α,2α-epoxy-4,6-androstadiene-3,17-dione (**2**) using HCl, HBr and NaN_3_, respectively. Compound **2** was also reacted with NaCN to give 2-cyano-1,4,6-androstatriene-3,17-dione (**5**) and 2β-cyano-1α-hydroxy-4,6-androstadiene-3,17-dione (**6**). 6α,7α-Epoxy-1,4-androstadiene-3,17-dione (**8**) was reacted with HCl, HBr and NaN_3_ to form the corresponding 7β-chloro-, 7β-bromo- and 7β-azido-6α-hydroxy-1,4-androstadiene-3,17-diones. The cytotoxic activity of these compounds towards T47D (estrogen-dependent) and MDA-MB231 (estrogen-independent) breast cancer cell lines was evaluated. The 6α-hydroxy-7β-substituted analogs were more active than the 2-substituted analogs on both cell lines. Compound **2** showed the highest selective activity against the T47D (IC_50_7.1 μM) cell line and **5** showed good cytotoxic activity on MDA-MB231 (IC_50_18.5 μM) cell line, respectively. The 6α,7α-epoxy analog **8** also showed high cytotoxic activity on both cell lines (IC_50_ 17.3 μM on T47D and IC_50_ 26.9 μM on MDA-MB231).

## 1. Introduction

Breast cancer is the most commonly diagnosed cancer among women and continues to be a major cause of cancer related deaths. The medical treatments available for breast cancer include endocrine therapy, cytotoxic chemotherapy and adjuvant treatments. Estrogens are involved in numerous physiological processes, including the development and maintenance of the female sexual organs, the reproductive cycle, reproduction, and various neuroendocrine functions. On the other hand, estrogens enhance growth and proliferation of certain target cells, such as breast epithelial cells and estrogen-dependent mammary carcinoma cells. Tamoxifen is an estrogen receptor antagonist in breast tissue and has been the standard endocrine (antiestrogen) therapy for hormone-sensitive early breast cancer in post-menopausal women, however, prolonged treatment may cause endometrial cancer [2]. Estrone and estradiol are biosynthesized from 4-androstene-3,17-dione (4-AD) and testosterone by aromatase and consequently compounds (e.g., exemestane, formestane and anastrozole) that inhibit the aromatase enzyme find application in the treatment of advanced estrogen-dependent breast cancers [3,4]. Locally advanced or metastatic breast cancer is typically treated with chemotherapy [5]. Cyclophospamide, methotrexate and fluorouracil have all been used as chemotherapeutic drugs to treat breast cancer. Currently available cytotoxic drugs do not discriminate between cancer and normal cells undergoing rapid division, although some antiestrogen and aromatase inhibitors offer a greater breadth of endocrine therapy and lower toxicity than some other drugs.

Dehydroepiandrosterone (DHEA), a precursor of 4-AD, is known to have chemopreventive and anti-proliferative actions on tumors [6,7]. DHEA retarded chemically induced carcinogenesis in liver [8], colon [9], lung [10], and breast [11]. 6-Hydroxyiminoandrostenedione was reported to cause a significant decrease in the growth of T47D breast cancer cells [12]. Sadekova *et al*. determined and characterized the expression of aromatase mRNA in the breast carcinoma cell lines T47D and MCF-7 [13]. Several hydroxyiminosteroid analogs have been synthesized and evaluated for their cytotoxic activities against several types of cancer cells such as MCF-7 and MDA-MB231, P-388 (murine leukemia), A-549 (human lung carcinoma), HT-29 (human colorectal adenocarcinoma) and MEL-28 (human myeloma) tumor cells [14,15,16]. Djurendić *et al*. described the synthesis of some 17α-picolyl- and 17-picolylideneandrost-5-ene derivatives and their antiaromatase and antitumor activity against the ER+ human breast adenocarcinoma MCF-7, and ER- human breast adenocarcinoma MDA-MB231, and PC-3 prostate cancer cell lines. 4-Hydroxy-17(*Z*)-picolinylidene-4,6-androstadien-3-one showed markedly strong cytotoxicity (IC_50_ 9.3 μM) against the MDA-MB231 cancer cell line, while also exhibiting a pronounced aromatase inhibition (92%). Formestane was reported to exhibit weak cytotoxic activity against MDA-MB231 (IC_50_ 55.5 μM) and no cytotoxic activity against the MCF-7 cell line [17,18], while 4-amino-4,6-androstadiene-3,17-dione, an analog of formestane, showed *in vitro* activity against the MCF-7 breast cancer cell line [19]. Androstene-fused *p*-methoxyphenyl-hydrazoline displayed the highest cytotoxic activity against MCF-7 (IC_50_ 2.16 μM), which was better than that of cisplatin (IC_50_ 9.63 μM) [20]. Steroidal endocyclic dienes and trienes have also been reported to show high biological activity [21,22]. 

We describe here the synthesis of some 2-substituted androstatriene- and 6α-hydroxy-7β-substituted androstadiene-3,17-dione derivatives which were modified with unsaturation in the A and/or B rings. Chloro, bromo, cyano, and azido groups were selected as substituents at the C-2 and C-7 positions in the androstane skeleton. The *in* *vitro* cytotoxic activity of these substituted androstadiene and androstatriene derivatives against estrogen-dependent (T47D) and estrogen-independent (MDAMB231) breast cancer cells was studied.

## 2. Results and Discussion

### 2.1. Synthesis

**Scheme 1:**
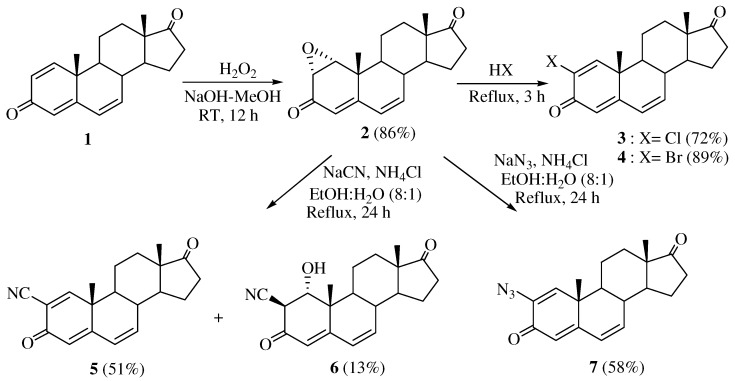
Synthesis of 2-substituted-1,4,6-androstatriene-3,17-diones.

The synthetic route to the 2-substituted 1,4,6-androstatriene-3,17-diones 3-7 is shown in Scheme 1. 1,4,6-Androstatriene-3,17-dione (**1**) was synthesized from DHEA with 2,3-dichloro-4,5-dicyano-1,4-benzoquinone (DDQ) by the previously reported method [23]. The starting material 1α,2α-epoxy-4,6-androstadiene-3,17-dione (**2**) as obtained stereoselectively by reacting **1** with hydrogen peroxide in 5% NaOH-MeOH. 2-Chloro- and 2-bromo-1,4,6-androstatriene-3,17-diones **3** and **4** were obtained from **2** by refluxing with concentrated HCl and HBr [24].

When **2 **wasre fluxed with sodium cyanide and ammonium chloride in 8:1 ethanol-H_2_O solution for 24 h, 2-cyano-1,4,6-androstatriene-3,17-dione (**5**) was obtained in 51% yield, along with 2β-cyano-1α-hydroxy-4,6-androstadiene-3,17-dione (**6**, 13%) as a side product. The ^1^H-NMR spectrum of **5** showed peaks for four double bond hydrogens at δ 7.01 (H-1), 6.30 (H-7), 6.22 (H-6) and 5.90 (H-4) ppm. Six double bond carbons and the CN carbon were assigned to the peaks at 163.7, 160.5, 139.0, 127.7, 124.1, 118.1 and 116.3 (CN) ppm in the ^13^C-NMR spectrum and the mass spectrum indicated a molecular ion [M]^+^ at m/z 307. The ^1^H-NMR spectrum of **6** showed peaks at 6.28 (H-7), 6.19 (H-6) and 5.68 (H-4) ppm for three double bond hydrogens and at δ 4.17 and 3.51 ppm for H-1 and H-2, respectively. Peaks at 155.8, 143.0, 132.2 and 123.7 ppm in the ^13^C-NMR spectrum were assigned to four double bond carbons and the CN carbon was identified at 121.3 ppm. The stereochemistry of H-1 and H-2 were determined with the 1D-NOESY spectrum. Thus, the β-configuration of H-1 was confirmed by irradiation of the H-1 proton which showed a NOE at H-19. The α-configuration of H-2 was also identified by irradiation of the H-2 proton which did not show a NOE at H-19. 2-Azido-1,4,6-androstatriene-3,17-dione (**7**) was synthesized and identified by the same method. 

**Scheme 2:**
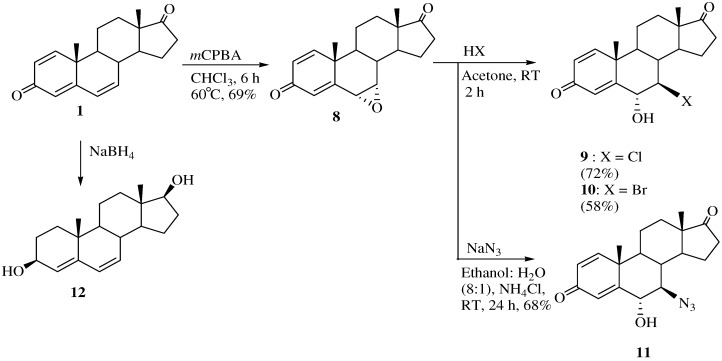
.Synthesis of 6α-hydroxy-7β-substituted-1,4-androstatriene-3,17-diones.

The synthetic routes to the 6α-hydroxy-7β-substituted 1,4-androstadiene-3,17-diones are shown in Scheme 2. To synthesize the 6α-hydroxy-substituted analogs, the reactions were run at room temperature. 6α,7α-Epoxy-4,6-androstadiene-3,17-dione (**8**) was stereoselectively obtained by reacting **1** with *m*-chloroperoxybenzoic acid [20].

7β-Chloro- and 7β-bromo-6α-hydroxy-1,4-androstadiene-3,17-diones **9** and **10** were obtained from **8** by treatment with concentrated HCl or HBr at room temperature for 2 h. The ^1^H-NMR spectrum of **9** showed peaks at δ 7.49 (H-1), 6.43 (H-7), 6.27 (H-6), and 6.17 (H-4) ppm for the four double bond hydrogens in the steroid A ring. The four double bond carbons of **9** were assigned to the peaks at 160.4 (C-5), 157.8 (C-1), 131.0 (C-2) and 126.6 (C-4) ppm in the corresponding ^13^C-NMR spectrum and the mass spectrum indicated a molecular ion [M]^+^ at m/z 334. Compound **8** was reacted with sodium azide in ethanol/H_2_O (1:8) at room temperature to form 7β-azido-6α-hydroxy-1,4-androstadiene-3,17-dione (**11**) in 68% yield. The stereochemistry of H-6 and H-7 were determined with the aid of the corresponding 1D-NOESY spectrum. The β-configuration of H-6 was confirmed by irradiation of the H-6 proton which showed a NOE on the H-19 protons and the α-configuration of H-7 was also identified by irradiation of the H-19 protons which did not show a NOE on the H-19 protons. 4,6-Androstadiene-3β,17β-diol (**12**) was obtained from the reaction of **1** and sodium borohydride [21]. The stereochemistry of the 3- and 17-hydroxy groups was determined from the 1D-NOESY spectrum. The 3β-hydroxy configuration was confirmed by selective irradiation of the H-19 methyl hydrogens which yielded no enhancement of the H-3 signal. In addition, since the NMR signal for H-4 is a sharp singlet at 5.88 ppm (not coupled with H-3), the dihedral angle between H-3 and H-4 must be close to 90 degree (Karplus equation), implying that H-3 has an α-configuration. The 17β-hydroxy configuration was also confirmed by irradiation of the H-17 proton which did not show a NOE on the H-18 protons.

**Scheme 3:**
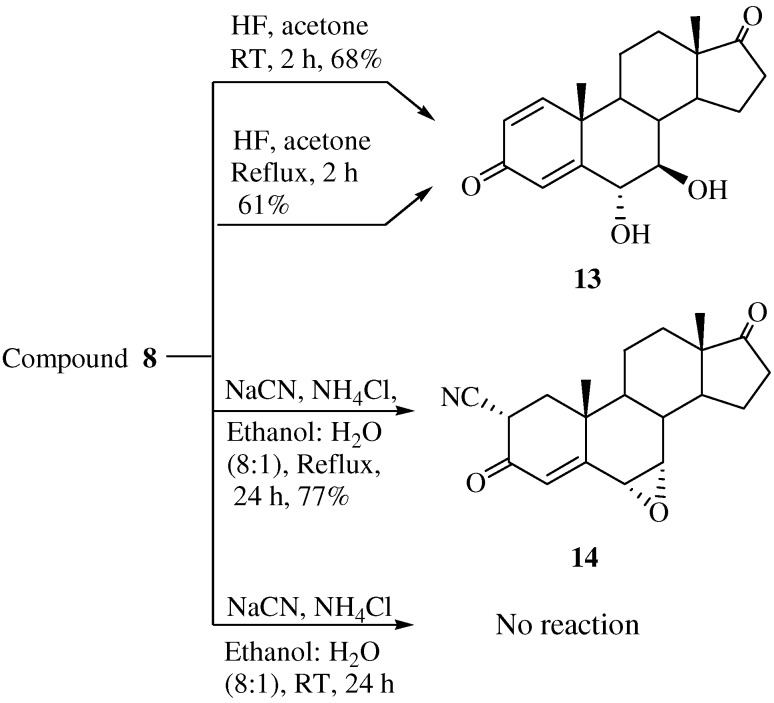
Synthesis of compounds **13** and **14**.

Unexpectedly, when compound **8** was treated with commercially available HF both at room temperature and at high temperature 6α,7β-dihydroxy-1,4-androstadiene-3,17-dione (**13**) was obtained instead of the expected compound, 7β-fluoro-6α-hydroxy-1,4-androstadiene-3,17-dione (Scheme 3). Two hydroxyl peaks were identified as a broad singlet at δ 5.43 and 4.72 ppm in the ^1^H-NMR spectrum and two hydroxyl carbon peaks at 71.7 (C-6) and 63.2 (C-7) ppm were identified in the ^13^C- NMR spectrum. The mass spectrum of compound **13 **showed a molecular ion [M]^+^ at m/z 316 (C_19_H_24_O_4_). The stereochemistry of H-6 and H-7 were determined from the 1D-NOESY spectrum. The α-configuration of OH-6 was confirmed by irradiation of the H-6 proton, which showed a NOE on the H-19 protons. The β-configuration of OH-7 was also verified by irradiation of the H-7 proton which did not show a NOE on the H-19 protons.

**Figure 1:**
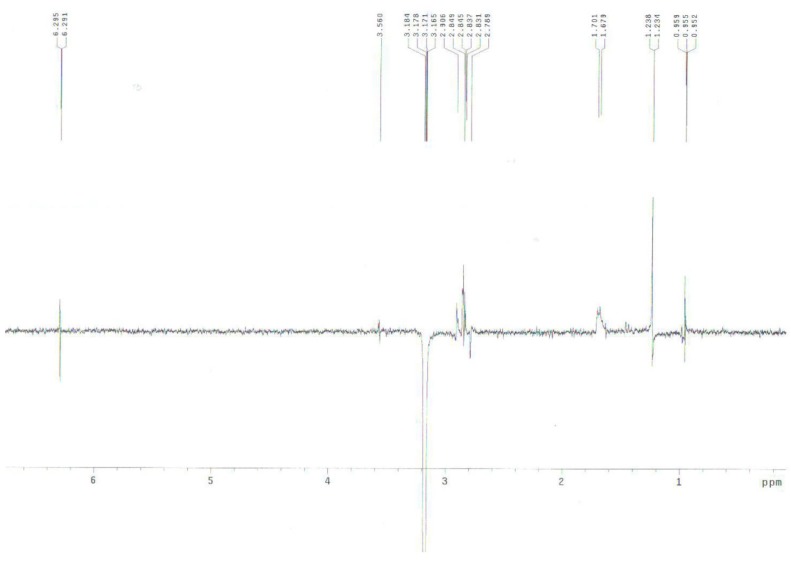
1D-NOESY spectrum of compound **14**.

**Figure 2:**
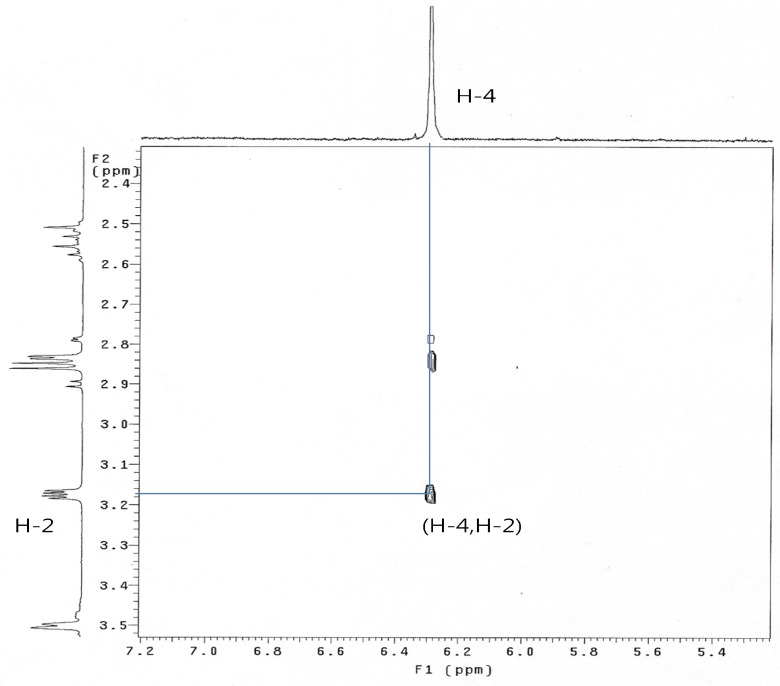
^1 ^H-^1^H COSY spectrum of compound **14**.

When **8 **was treated with sodium cyanide and ammonium chloride in ethanol/H_2_O (8:1) solution, the reaction did not proceed at room temperature, but when **8** was reacted under reflux conditions, 2α-cyano-6α,7α-epoxy-4-androstene-3,17-dione (**14**) was obtained in 77% yield. The structure of **14** was determined by the presence of peaks at δ 6.29 ppm for only one double bond hydrogen (H-4) and at 3.17 (H-2) ppm and at δ 3.56 (H-7) and 3.49 (H-6) ppm for two epoxy hydrogens on the ^1^H-NMR spectrum and two double bond carbons at 158.6 (C-5), 131.8 (C-4) and a CN carbon at 118.5 ppm, and two epoxy carbons at 53.3 and 52.5 ppm in the ^13^C-NMR spectrum. The mass spectrum of compound **14** showed a molecular ion [M]^+^ at m/z 325 (C_20_H_2__3_NO_3_). The configuration and position of cyano group were determined from the 1D-NOESY and ^1^H-^1^H COSY data. The α-configuration of the CN substituent was identified by irradiation of the H-2 proton which showed a NOE on the H-19 protons in the 1D-NOESY spectrum (Figure 1). We acquired the correlation information between H-2 and H-4 from the cross peak observed in the ^1^H-^1^H COSY spectrum (Figure 2).

### 2.2. Cytotoxic effects

**Figure 3:**
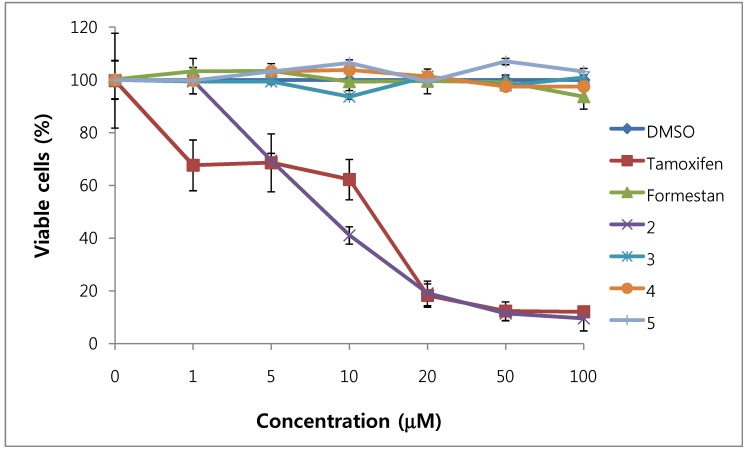
Cytotoxic effects of compounds **2–5** in T47D cells (ER-positive breast cancer cells).

**Figure 4:**
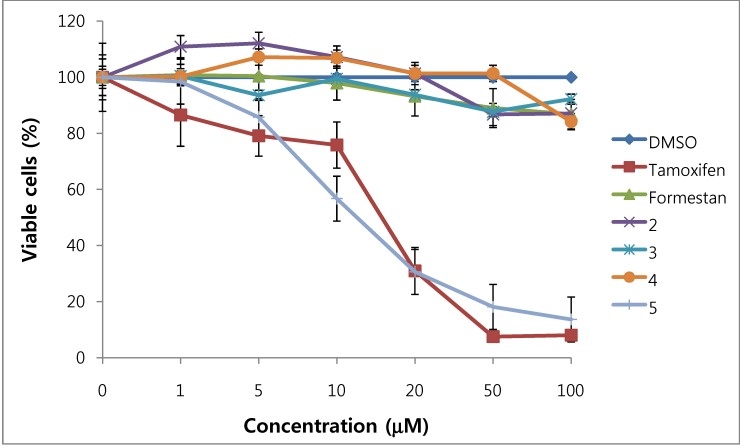
Cytotoxic effects of compounds **2 - 5** in MDA-MB231 cells (ER-negative breast cancer cells).

**Figure 5:**
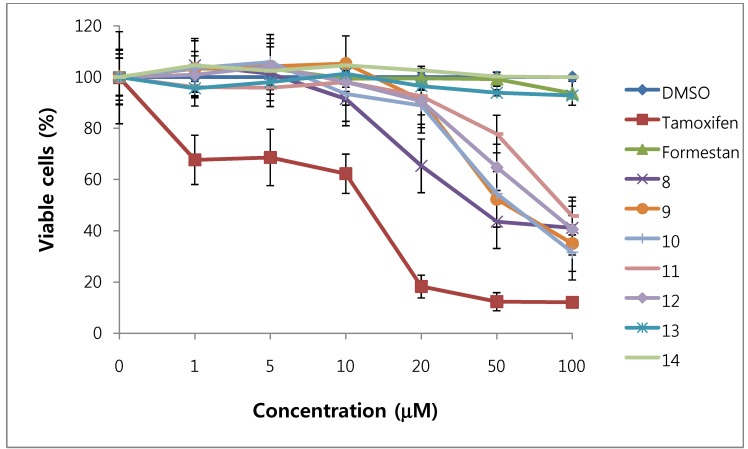
Cytotoxic effects of compounds **8–14** in T47D cells (ER-positive breast cancer cells).

**Figure 6:**
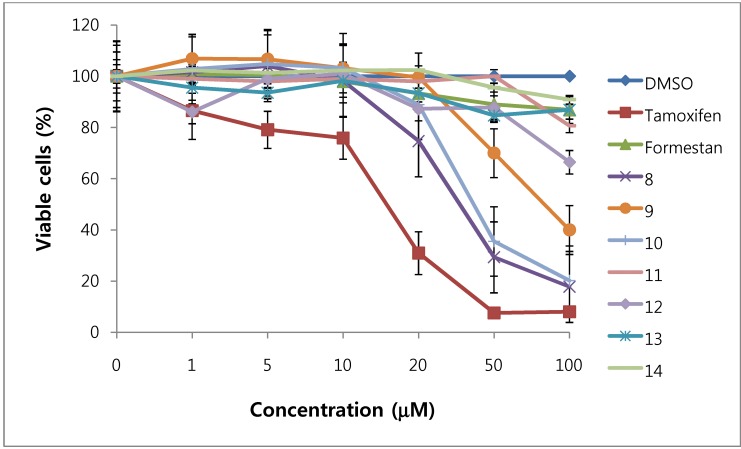
Cytotoxic effects of compounds **8 - 14** in MDA-MB231cells (ER-negative breast cancer cells).

We assessed the cytotoxicity of synthesized compounds **1-5 **and **7-14 **on T47D and MDA-MB231 breast cancer cells. The results are shown in Figure 3, Figure 4, Figure 5, Figure 6 and Table 1. 

In general, 6α-hydroxy-7β-substituted analogs (Figure 5 and Figure 6) were more active than 2-substituted analogs (Figure 3 and Figure 4). The cytotoxicity of the synthesized compounds was slightly higher against estrogen-dependent breast cancer cells (T47D) than estrogen-independent breast cancer cells (MDA-MB231). While formestane did not show cytotoxicity towards either the T47D and MDA-MB231 breast cancer cell lines, tamoxifen displayed significantly high cytotoxicity on both. As shown in Figure 3 and Figure 4, 1α,2α-epoxy-4,6-androstadiene-3,17-dione (**2**) showed a selective dose dependent and high cytotoxicity on T47D cells (IC_50_7.1 μM), which was better than that of tamoxifen (IC_50_ 13.0 μM, Table 1) and cisplatin (IC_50_ 9.63 μM) [20], but was non-cytotoxic on MDA-MB231 cells. We thus presume that a 1α,2α-epoxy ring may be associated with cytotoxicity on estrogen-dependent breast cancer cells. 2-Cyano-1,4,6-androstatriene-3,17-dione (**5**) showed good cytotoxic activity with an IC_50_ value of 18.7 μM on estrogen-independent breast cancer cells (MDA-MB231). 7β-Chloro- and 7β-bromo-6α-hydroxy-1,4-androstadiene-3,17-dione (**9** and **10**), and 4.6-androstadiene-3,17-diol (**12**) showed weak or moderate cytotoxic activity against these two cell lines, but 6α,7β-dihydroxy derivative **13** did not exhibit any cytotoxic activity against both cell lines. 6α,7α-Epoxy-1,4-androstadiene-3,17-dione (**8**) also showed high cytotoxic activity against both cell lines (IC_50_ 17.3 μM on T47D and IC_50_ 26.9 μM on MDA-MB231 cell lines) (Figure 5 and Figure 6) and the cytotoxic ability of **8 **seemed to be due to epoxy ring on 6,7-positions of steroid structure regardless of cell type. Unlike the 2-cyano analog **5 **and 6α,7α-epoxy analog **8**, 2α-cyano-6α,7α-epoxy-4-androstene-3,17-dione (**14**) exhibited no cytotoxic activity against either of these two cell lines.

**Table 1:** The IC_50_ values of the synthesized compounds on T47D and MDA-MB231cell lines.

Compound No.	T47D cells	MDA-MB231 cells
	IC_50_ (μM)	IC_50_ (μM)
Formestane	96.5	>100
Tamoxifen	13.0	11.4
**2**	7.1	>100
**3**	>100	>100
**4**	>100	>100
**5**	>100	18.7
**7**	>100	>100
**8**	17.3	26.9
**9**	36.4	53.5
**10**	44.2	33.1
**11**	95.6	>100
**12**	54.3	43.8
**13**	>100	>100
**14**	97.1	>100

## 3. Conclusions

Fourteen 2-substituted 1,4,6-androstatriene-3,17-diones and 6*α*-hydroxy-7β-substituted-1,4-androstadiene-3,17-diones were synthesized and twelve compounds were evaluated for cytotoxicity on T47D (ER-dependent) and MDA-MB231(ER-independent) cancer cell lines. 6*α*-Hydroxy-7β-substituted derivatives were more active than 2-substituted analogs against both cell lines. The cytotoxicity of the synthesized compounds was slightly higher on estrogen dependent breast cancer cells (T47D). The 1*α*,2*α*-epoxy compound **2** showed the highest cytotoxic effect on T47D (IC_50_ 7.1 μM) breast cancer cells. Compound **5** showed good cytotoxic activity on MBA-MB231 breast cancer cells (IC_50_ 18.7 μM) and the 6α,7α-epoxy analog **8** also showed high cytotoxic activity on both cell lines.

## 4. Experimental

### 4.1. General

All non-aqueous reactions were performed under an atmosphere of dry nitrogen. The commercial reagents were purchased from Aldrich, Fluka, or Sigma. Solvents were purified and dried prior to use. Melting points were measured on Thomas-Hoover melting point apparatus and not corrected. ^1^H-, ^13^C- NMR, HSQC, HMQC and NOESY spectra were recorded on a Varian 400 MHz spectrometer in CDCl_3_ and DMSO-*d_6_*. Chemical shifts (δ) are in parts per million (ppm) relative to tetramethylsilane, and coupling constants (*J*) are in Hertz. IR spectra were determined on a Jasco FT-IR 300E spectrometer as KBr pellets, unless indicated otherwise. GC/MS spectra were obtained on a Shimadzu QP 5050 and JEOL GC Mate 2 mass spectrometers. MPLC was run on a Yamazen YFLC-AI instrument. Analytical TLC was performed on pre-coated silica gel 60 F_254_ plates (Merck). Solvent systems for TLC were ethyl acetate/*n*-hexane mixtures and 10% methanol in dichloromethane. Column chromatography was carried out on Merck silica gel 9385 (230-400 mesh), eluting with ethyl acetate/*n*-hexane mixtures.

### 4.2. Synthesis of 2-substituted-1,4,6-androstatriene-3,17-dione derivatives

*1α,2α-Epoxy-4,6-androstadiene-3,17-dione* (**2**): According to the previously reported procedure [25], 1,4,6-androstatiene-3,17-dione (**1**, 1g, 3.5 mmol) was dissolved in methanol (15 mL), then 5% NaOH-MeOH (2.5 mL) and 30% H_2_O_2_(12.5 mL) were added and the mixture was stirred at room temperature for 12 h. Yield: 86% (900 mg), mp: 219-220 °C (lit. [25] 220–221 °C).

*2-Chloro*-*1,4,6-androstatriene-3,17-dione* (**3**): Compound **2** (100 mg, 0.34 mmol) in ethanol (5 mL) was stirred with concentrated HCl (0.46 mL) at room temperature and then refluxed for 3 h. The reaction mixture was neutralized with 10% NaOH to give a pale yellow compound, which was recrystallized with dichloromethane and *n*-hexane to afford pure white product **3**. Yield: 72% (77 mg), mp: 242–243 °C (lit. [24] 240–242 °C). 

*2-Bromo-1,4,6-androstatriene-3,17-dione* (**4**): Compound **2** (100 mg, 0.34 mmol) in ethanol (5 mL) was stirred with concentrated HBr (0.2 mL) at room temperature and the reaction mixture was then refluxed for 3 h and treated as the same method of compound **3 **to give a pure white product **4**. Yield: 89% (109 mg), mp: 267–268 °C (lit. [24] 265–267 °C).

*2-Cyano-1,4,6-androstatriene-3,17-dione* (**5**) *and 2β-cyano-1α-hydroxy-4,6-androstadiene-3,17-dione* (**6**): Compound **2** (200 mg, 0.68 mmol) was dissolved in 8:1 ethanol-H_2_O (9 mL) and NH_4_Cl (71 mg, 1.32 mmol) and NaCN (162 mg, 3.31 mmol) were added and the resulting mixture was refluxed for 24 h. The reaction mixture was neutralized with 10% HCl in ice bath, extracted with dichloromethane (30 mL× 3) and washed with water (30 mL). The combined organic layers were dried with anhydrous magnesium sulfate, filtered, and concentrated to give a crude oil, which was purified by column chromatography (ethyl acetate-*n*-hexane = 1:3) to give pure white compound **5** and oily compound **6**. Compound **5**: yield: 51% (105 mg), mp: 185–186 °C; IR (cm^-1^): 2950, 2251 (CN), 1735 (17-C=O), 1653 (3-C=O); ^1^H-NMR (DMSO-*d_6_*) δ: 7.01 (1H, s, H-1), 6.30 (1H, d, *J =* 10.0 Hz, H-7), 6.22 (1H, d, *J* = 10.4 Hz, H-6), 5.90 (1H, s, H-4), 1.03 (3H, s, H-19), 0.84 (3H, s, H-18); ^13^C- NMR (DMSO-*d_6_*) δ: 218.6 (C-17), 173.9 (C-3), 163.7 (C-5), 160.5 (C-1), 139.0 (C-7), 127.7 (C-6), 124.1 (C-4), 118.1 (C-2), 116.3 (CN), 49.1, 48.3, 46.9, 44.3, 38.6, 35.7, 32.5, 24.2, 21.4, 20.1, 13.1, GC-MS (EI) m/z: 307 [M]^+^. Compound **6**: yield: 13% (30 mg); IR (neat, cm^-1^): 3358 (OH), 2945, 2249 (CN), 1729 (17-C=O), 1644 (3-C=O); ^1^H-NMR (DMSO-*d_6_*) δ: 6.28 (1H, d, *J =* 9.8 Hz, H-7), 6.19 (1H, d, *J* = 9.2 Hz, H-6), 5.68 (1H, s, H-4), 4.17 (1H, d, *J* = 3.8 Hz, H-1), 3.51 (1H, d, *J* = 3.6 Hz, H-2), 0.98 (3H, s, H-19), 0.83 (3H, s, H-18); ^13^C-NMR (DMSO-*d_6_*) δ: 224.0 (C-17), 180.1 (C-3), 155.8 (C-5), 143.0 (C-7), 132.2 (C-6), 123.7 (C-4), 121.3 (CN), 77.2 (C-1), 53.8 (C-2), 52.8, 52.5, 41.7, 41.0, 40.5, 36.3, 36.2, 25.2, 20.0, 18.7, 13.4; GC-MS (EI) m/z: 325 [M]^+^, 307 [M-H_2_O]^+^.

*2-Azido-1,4,6-androstatriene-3,17-dione* (**7**): Compound **2** (200 mg, 0.68 mmol) was dissolved in 8:1 ethanol-H_2_O (9 mL) and NH_4_Cl (71 mg, 1.33 mmol) and NaN_3_ (215 mg, 3.31 mmol) were added and the mixture refluxed for 24 h. The reaction mixture was cooled down and treated with NH_4_Cl to obtain a crude brown compound, which was extracted with dichloromethane (30 mL × 3) and washed with water (30 mL). The organic layer was dried with anhydrous magnesium sulfate, filtered, and concentrated to crude oil, which was purified by column chromatography (ethyl acetate-*n*-hexane=1:3) to give pure white compound **7**. Yield: 58% (126 mg), mp: 248–249 °C; IR (cm^-1^): 2948, 1721 (17-C=O), 1640 (3-C=O); ^1^H-NMR (CDCl_3_) δ: 6.50 (1H, s, H-1), 6.33 (1H, d, *J* = 12.4 Hz, H-7), 6.16 (1H, d, *J* = 10.0 Hz, H-6), 6.06 (1H, s, H-4), 1.24 (3H, s, H-19), 1.00 (3H, s, H-18); ^13^C-NMR (CDCl_3_) δ: 218.9 (C-17), 181.0 (C-3), 162.6 (C-5), 136.8 (C-1), 134.9 (C-2), 134.4 (C-7), 127.8 (C-6), 123.0 (C-4), 48.9, 48.8, 47.8, 41.6, 37.5, 35.5, 31.2, 21.3, 21.2, 13.8; GC-MS (EI) m/z: 295 [M-N_2_]^+^. 

### 4.3. Synthesis of 7-substituted-1,4-androstadiene-3,17-dione derivatives

*1α,2α-Epoxy-4,6-androstadiene-3,17-dione* (**8**): According to the previously reported procedure [23], 1,4,6-androstatriene-3,17-dione (**1**, 1 g, 3.5 mmol) was dissolved in chloroform (15 mL) and *m*-chloroperoxybenzoic acid (980 mg, 5.7 mmol) was added and stirred at 60 °C for 6 h. Yield: 61% (640 mg), mp : 199–202 °C (lit. [23] 200–203 °C). 

*7β-Chloro-6 α-hydroxy -1,4-androstadiene-3,17-dione* (**9**): Compound **8** (100 mg, 0.34 mmol) was dissolved in acetone (2 mL), HCl (0.46 mL) was added at 0 °C and the mixture stirred at room temperature for 2 h. The reaction mixture was neutralized with 10% NaOH to form a pale yellow precipitate. The crude precipitate was filtered and washed with water and purified by column chromatography (ethyl acetate-*n*-hexane=1:5) to give pure white compound **9**. Yield: 72% (82 mg), m.p: 265–267 °C; IR (cm^-1^): 3346 (OH), 2958, 1737 (17-C=O), 1656 (3-C=O); ^1^H-NMR (CDCl_3_) δ: 7.24 (1H, d, *J* = 10.0 Hz, H-1), 6.34 (1H, d, *J* = 2.0 Hz, H-4), 6.15 (1H, dd, *J* = 2.0, 9.6 Hz, H-2), 4.76 (1H, d, *J* = 2.8 Hz, H-6), 3.91 (1H, t, *J* = 4.8 Hz, H-7), 1.40 (3H, s, H-19), 0.89 (3H, s, H-18); ^13^C-NMR (CDCl_3_) δ: 219.8 (C-17), 185.5 (C-3), 160.4 (C-5), 157.8 (C-1), 131.0 (C-2), 126.6 (C-4), 71.7 (C-6), 63.2 (C-7), 47.4, 45.1, 43.5, 42.2, 35.8, 34.1, 31.4, 23.0, 21.5, 21.4, 13.9; GC-MS (EI) m/z: 334 [M]^+^.

*7β-Bromo-6α-hydroxy-1,4-androstadiene-3,17-dione* (**10**)*:* Compound **8** (100 mg, 0.34 mmol) was dissolved in acetone (2 mL), HBr (0.2 mL) was added at 0 °C and the mixture stirred at room temperature for 2 h. and then treated by the same method of compound **9 **to give a pure white product **10**. Yield: 58% (75 mg), m.p: 225–227 °C; IR (cm^-1^): 3356 (OH), 2950, 1739 (17-C=O), 1651 (3-C=O); ^1^H-NMR (CDCl_3_) δ: 7.24 (1H, d, *J* = 10.4 Hz, H-1), 6.38 (1H, d, *J* = 2.0 Hz, H-4), 6.15 (1H, dd, *J* = 2.0, 14.0 Hz, H-2), 4.91 (1H, d, *J* = 2.4 Hz, H-7), 3.99 (1H, d, *J* = 2.6 Hz, H-6), 1.46 (3H, s, H-19), 0.90 (3H, s, H-18); ^13^C-NMR (CDCl_3_) δ: 219.7(C-17), 185.4 (C-3), 160.9 (C-5), 157.6 (C-1), 130.7 (C-2), 126.5 (C-4), 72.0 (C-6), 54.2 (C-7), 47.3, 43.7, 41.7, 35.8, 34.3, 31.4, 24.4, 21.5, 21.3, 13.9, 45.3; GC-MS (EI) m/z: 379 [M]^+^.

*7β-Azido-6α-hydroxy-1,4-androstadiene-3,17-dione* (**11**)*:* Compound **8** (200 mg, 0.67 mmol) was dissolved in 8:1 ethanol-H_2_O (9 mL), NH_4_Cl (71 mg, 1.32 mmol) and NaN_3_ (215 mg, 3.3 mmol) were added and the mixture refluxed for 24 h. The reaction mixture was then neutralized with 10% HCl to give a yellow precipitate which was filtered, washed with water and purified by column chromatography (ethyl acetate-*n*-hexane=1:3) to give a pure pale yellow compound **11**. Yield: 68% (155 mg), m.p: 275–277 °C, IR (cm^-1^): 3348 (OH), 2959, 1730 (17-C=O), 1649 (3-C=O); ^1^H-NMR (CDCl_3_) δ: 7.21 (1H, d, *J* = 10.4 Hz, H-1), 6.29 (1H, d, *J* = 1.6 Hz, H-4), 6.13 (1H, dd, *J* = 1.6, 10.0 Hz, H-2), 5.29 (1H, d, *J* = 1.6 Hz, H-4), 4.47 (1H, d, *J* = 2.8 Hz, H-6), 3.65 (1H, d, *J* = 1.4 Hz, H-7), 1.31 (3H, s, H-19), 0.83 (3H, s, H-18); ^13^C-NMR (CDCl_3_) δ: 224.6 (C-17), 190.0 (C-3), 164.6 (C-5), 162.3 (C-1), 135.9 (C-2), 131.6 (C-4), 74.0 (C-6), 52.1, 49.8, 47.8, 47.2, 40.5, 39.8, 36.1, 26.2, 26.1, 25.1, 18.6; GC-MS (EI) m/z: 313 [M-N_2_]^+^.

*4,6-Androstadiene-3β,17β-diol* (**12**)*:* A solution of compound **8** (100 mg, 0.34 mmol) in ethanol (30 mL) and sodium borohydride (53 mg, 1.4 mmol) was stirred at room temperature for 4 h. The reaction mixture was evaporated to form an oily residue which was treated with H_2_O and then with 10% HCl, H_2_O and saturated NaHCO_3 _and extracted with dichloromethane. The organic layer was dried with anhydrous magnesium sulfate, filtered, and concentrated to a crude oil, which was purified by column chromatography (ethyl acetate-*n*-hexane=1:3) to give pure white compound **12**. Yield: 69% (695 mg), m.p : 157–158 °C (lit. [23] 155–157 °C). 

*6 α,7β -Dihydroxy-1,4-androstadiene-3,17-dione* (**13**): Compound **8** (100 mg, 0.34 mmol) was dissolved in acetone (2 mL) and HF (0.3 mL) was added at 0 °C and stirred at room temperature for 2 h and treated as the same method of compound **9 **to give a pale yellow product which was purified by column chromatography (ethyl acetate-*n*-hexane=1:1) to give pure white compound **13**. Yield: 68% (71 mg), m.p: 280–282 °C, IR (cm^-1^): 3350 (OH), 2952, 1734 (17-C=O), 1653 (3-C=O); ^1^H-NMR (CDCl_3_) δ: 7.11 (1H, d, *J* = 12.4 Hz, H-1), 6.04 (1H, d, *J* = 2.0, 10.0 Hz, H-2), 5.97 (1H, s, H-4), 5.43 (1H, br s, C_6_-OH), 4.72 (1H, br s, C_7_-OH), 4.08 (1H, s, H-7), 3.69 (1H, s, H-6), 1.29 (3H, s, H-19), 0.82 (3H, s, H-18); ^13^C-NMR (CDCl_3_) δ: 220.1 (C-17), 185.9 (C-3), 166.0 (C-5), 158.2 (C-1), 128.1 (C-2), 126.6 (C-4), 77.6 (C-6), 71.6 (C-7), 55.6, 47.5, 45.2, 43.7, 43.6, 35.9, 34.6, 31.5, 31.4, 21.7, 21.6, 20.9, 13.8; GC-MS (EI) m/z: 316 (M)^+^.

2α*-Cyano-6α,7α-epoxy-4-androstene-3,17-dione* (**14**)*:* Compound **8** (200 mg, 0.67 mmol) was dissolved in 8:1 ethanol-H_2_O (9 mL), NaCN (162 mg, 3.30 mmol) was added and the mixture was stirred at room temperature for 4 h. The reaction mixture was cooled down and treated like compound **11 **to give a pure pale yellow product **14**. Yield: 77% (167 mg), m.p : 286–288 °C, IR (cm^-1^): 2951, 2250 (CN), 1733 (17-C=O), 1649 (3-C=O), ^1^H-NMR (CDCl_3_) δ: 6.29 (1H, s, H-4), 3.56 (1H, d, *J =* 3.6 Hz, H-7), 3.49 (1H, d, *J* = 8.0 Hz, H-6), 3.17 (1H, dd, *J =* 2.8,5.2 Hz, H-2), 1.24 (3H, s, H-19), 0.96 (3H, s, H-18); ^13^C-NMR (CDCl_3_) δ: 218.7 (C-17), 192.4 (C-3), 158.6 (C-5), 131.8 (C-4), 118.5 (CN), 53.3 (C-6), 52.5 (C-7), 47.9, 46.8, 38.4, 38.2, 36.8, 35.9, 35.8, 34.6, 31.0, 21.6, 19.3, 18.3, 13.8; GC-MS (EI) m/z: 325 [M]^+^.

### 4.4. Biology

T47D (estrogen receptor dependent cell line) and MDA-MB231 (estrogen receptor independent cell line) breast cancer cells were purchased from the Korean Cell Line Bank (Seoul). Formestane and tamoxifen were purchased from Aldrich and were used as positive controls. RPMI medium 1640, fetal bovine serum (FBS, Gibco BRL, Rockville, MD, USA), penicillin-streptomycin, phosphate buffered saline (PBS) pH 7.4, 0.25% trypsin EDTA, dimethylsulfoxide (DMSO, Sigma), thiazolyl blue tetrazolium bromide (MTT, Sigma) were also all commercially available reagents.

### MTT assay on T47D and MDA-MB231 cells

T47D and MDA-MB231 breast cancer cells were grown in RPMI medium 1640 containing 10% fetal bovine serum (FBS) supplemented with 1% penicillin-streptomycin. Cells (2 × 10^3^) were plated in 96 well plates and incubated in 5% CO_2_ incubator (NAPCO Water-Jacketed CO_2_ Incubator) for 24 h. Then cells were treated with 0, 1, 5, 10, 20, 50, 100 μM concentrations of sample (5 μL/well) and incubated in 5% CO_2_ incubator for 48 h at 37 °C. Triplicate wells were used for each concentration. After incubation for 48 h, 50 μL/well of MTT (2 mg/mL in PBS) was added and further incubated for 4 h in the dark. After removal of the MTT solution, 150 μL/well DMSO was added to dissolve the purple crystal for 15 minutes, after which the absorbance of the solution was measured at 540 nm with an ELISA reader (ELx 808, BIO-TEK). The inhibition effect of T47D and MDA-MB231 cell growth was expressed as relative cell survival percentage. Experiments were performed in triplicate.
